# The Proteasix Ontology

**DOI:** 10.1186/s13326-016-0078-9

**Published:** 2016-06-04

**Authors:** Mercedes Arguello Casteleiro, Julie Klein, Robert Stevens

**Affiliations:** School of Computer Science, University of Manchester, Oxford Road, Manchester, M13 9PL UK; Institut National de la Sante et de la Recherche Medicale (INSERM), U1048, Toulouse, 24105 France

**Keywords:** Ontology, Proteasix, Protease prediction, Peptide, Cleaveage site, Open biomedical ontologies

## Abstract

**Background:**

The Proteasix Ontology (PxO) is an ontology that supports the Proteasix tool; an open-source peptide-centric tool that can be used to predict automatically and in a large-scale fashion *in silico* the proteases involved in the generation of proteolytic cleavage fragments (peptides)

**Methods:**

The PxO re-uses parts of the Protein Ontology, the three Gene Ontology sub-ontologies, the Chemical Entities of Biological Interest Ontology, the Sequence Ontology and bespoke extensions to the PxO in support of a series of roles: 1. To describe the known proteases and their target cleaveage sites. 2. To enable the description of proteolytic cleaveage fragments as the outputs of observed and predicted proteolysis. 3. To use knowledge about the function, species and cellular location of a protease and protein substrate to support the prioritisation of proteases in observed and predicted proteolysis.

**Results:**

The PxO is designed to describe the biological underpinnings of the generation of peptides. The peptide-centric PxO seeks to support the Proteasix tool by separating domain knowledge from the operational knowledge used in protease prediction by Proteasix and to support the confirmation of its analyses and results.

**Availability:**

The Proteasix Ontology may be found at: http://bioportal.bioontology.org/ontologies/PXO. This ontology is free and open for use by everyone.

**Electronic supplementary material:**

The online version of this article (doi:10.1186/s13326-016-0078-9) contains supplementary material, which is available to authorized users.

## Background

Proteases are enzymes that catalyze peptide bond cleavage and this activity can lead to the generation of protein cleavage fragments or peptides. Proteases have a wide spectrum of specificity [1]. The human genome encodes over 550 different proteases, participating in many different biological processes, including protein degradation, immunity response, regeneration or cell division and are involved in diseases such as cancer, inflammation and cardiovascular disease [2, 3].

Body fluids (e.g. serum, urine, cerebrospinal fluid) contain thousands of protein fragments and disease-associated peptides. The proteolytic mechanisms that lead to the generation of these fragments may be associated with diseases, and are not well described in the literature. Further insight into the proteases implicated in peptide generation may help in understanding some diseases.

The particular function and specificity of each protease are defined by their binding to a characteristic amino acid motif that forms a cleavage site in the protein target [4]. Knowledge about proteases and their substrates and cleavage sites is scattered across publications and databases. These different resources do not permit cleavage site information to be retrieved from peptide sequence input automatically and thus elucidating the proteases implied in peptide production is difficult.

The Proteasix tool [5, 6], is an open-source peptide-centric tool that can be used to predict automatically the proteases involved in the generation of proteolytic cleavage fragments (peptides). Proteasix is a tool that uses protease/cleavage sites (CS) associations established by either observations or predictions to suggest the proteases implicated in the generation of a peptide. Proteasix does this by using the N- and C-terminal sequences of peptides that are reconstructed using information from the UniProt knowledge base [7] to identify the possible proteases that were involved in their generation [5]. Observations of protease/CS combinations were extracted from CutDB [8], UniProt and the literature. When a previous observation has not been established, Proteasix calculates the probability of protease/CS association by using the MEROPS [9] and BRENDA [10] databases. Proteases exhibit varying binding affinities for amino-acid sequences, ranging from strict restriction to one or few critical amino-acids in given positions, to generic binding with little discrimination between different amino acids [5].

The predictions currently made by Proteasix are agnostic as to the taxon of the organism whence the peptides come, the cellular location of the predicted proteases and the proteins they may cleave. Also, the function of the proteases, e.g., whether they are an endo- or exo-peptidase is not taken into account. This is the kind of knowledge an ontology is able to provide. Thus the new version of Proteasix uses the Proteasix Ontology (PxO) to make this knowledge available to its algorithm. We go on to describe the PxO and its role in Proteasix.

## Competency questions for the PxO

The PxO is written in the Web Ontology Language (OWL) [11] using the Protégé [12] version 5.0.0 beta 17 editor. In creating PxO we wished to undertake as little *de novo* ontology development as possible and to take advantage of the work already done in annotating gene products with the Gene Ontology (GO) [13]. This implied a strategy of re-using relevant Open biomedical Ontologies Consortium [14] (OBO) ontologies where possible, together with relevant annotations. The choice of which of the OBO to use was driven by a set of competency questions (CQ) that the PxO should fulfil. Once chosen, relevant portions of the ontologies were taken and extended in a way that accommodated the CQ, making appropriate commitments to the ontology used. The resulting PxO was then evaluated against the CQ.

To obtain the observed and predicted proteases responsible for the generation of peptides, the PxO needs to answer the following CQ:

1. What are the known protease and their target cleavage sites (observed and/or predicted)?
2. For a given peptide and protein from which it was derived, what are the cleavage sites that led to its production and is it the product of observed or predicted proteolysis?
3. What are the function, species and cellular location for both proteases and their substrate proteins?
4. For a given protease, what are its cleavage site specificity?
5. Given an amino acid, what are its biochemical properties?
6. For a protease predicted to have generated a peptide, what are its function and the processes in which it is known to participate?

The Additional file 1 provides the ELK reasoner times and shows the SPARQL SELECT queries for the CQ and the execution times for the CQ using JENA ARQ [15].

## Reuse of ontologies from OBO

To enable these competencies to be answered the PxO re-uses parts of some of the OBO; PxO uses the OWL [11] versions. After downloading the OWL files, a selection of class names (without deprecated classes); class expressions; class definitions; and annotation assertions were extracted. Where only a portion of the source ontology was required to support the CQ in PxO, we programmatically extracted a top-module [16]. A top-module is used as in the PxO only a restricted query supporting a CQ needs to be answered, rather than a query that necessitates all entailments from a signature to be preserved. The following OBO or their parts were used in PxO:

1. The Protein Ontology (PRO) [17] — Reuse of Protein(PR:000000001) and proteolytic cleavage product(PR:000018264) that are both subclasses of PRO’s amino acid chain (PR:000 018263). In order to follow the PRO annotation guidelines [18], the relationships participates in; located in; and has function were substituted with their Relationship Ontology equivalents. The use of the PRO supports all the CQ.
2. Relationship Ontology (RO) [19] — Where possible, PxO uses object properties from RO. For PxO, this includes has_function, has_location, participates_in and only_in_taxon.
3. the three Gene Ontology (GO) sub-ontologies [20] — First, a class name extraction was performed based on the three GO namespaces cellular component; molecular function; and biological process. As an example of usage in the PxO, the GO class peptidase activity (GO:0008233) was used to define protease molecular function, while proteolysis (GO:0006508) was used to describe the biological process of peptide production. Use of the GO supports CQ 1, 3 4 and 6.
4. Chemical Entities of Biological Interest Ontology (ChEBI) [21] — Reuse of chemical entity(CHEBI: 24431) that has as subclass molecular entity(CHEBI: 23367). PRO’s amino acid chain(PR:000018263) and amino acidwere made subclasses of ChEBI’s molecular entity(CHEBI:23367). The twenty amino acids are also taken from ChEBI. Some amino acids are interchangeable at a certain CS position, for they may have identical biochemical properties. This supports the answering of CQ 5.
5. Phenotypic Quality Ontology (PATO) [22] — Classes from PATO were reused to describe the properties of the amino acids. Hence, classes such as electric charge (PATO:0002193), polarity (PATO:0002182) as well as their superclasses like molecular quality (PATO:0002182) and subclasses such as negative charge (PATO:0002196) were extracted. The twenty amino acids from ChEBI are classified taken PATO’s descendants from molecular quality and side chain structure, which is outside of PATO. This helps to answer CQ 5.
6. the Sequence Ontology (SO) [23] — Cleavage site regions and C- and N-terminus of polypeptide sequences were described using polypeptide region (SO:0000839) to describe Cleavage site region. Moreover, the key classes to link proteins from Uniprot with gene names were described using gene (SO:0000704) along with its subclass protein coding gene (SO:0001217). Based on superkingdom and subclasses, i.e. upper-levels of the UniProtKB Taxonomy, the gene names are classified, and thereby, obtaining a hierarchy with three levels. These classes were used to support the CQ 1, 2, and 4.
7. The PRO proteins are organised based on taxon organism, and therefore, new classes under the PRO proteinclass were created according to the upper-levels of the UniProtKB Taxonomy [24]. These classes were used to support CQ 3.
8. GALEN ontology [25] — A medical ontology outside of OBO, which can be downloaded from BioPortal [26]. Reuse of the class KnowledgeStatus and the relationship hasKnowledgeStatus to represent observed or predicted proteolysis. To describe the level of confidence associated with a predicted proteolysis, the relationship hasConfidence LevelStatus the class ConfidenceLevel Status were also extracted. these classes were used to support CQ 1.

## PxO axioms and axiom patterns

***Peptide and protein***: A peptide, also known as proteolytic cleavage productin PRO, is described in the following way in PxO (all OWL fragments are represented using Manchester OWL Syntax [27]):



*Knowledge patterns* are *representations which capture recurring structure within and across ontologies* [28]. And therefore, *knowledge patterns* (patterns for short) can be seen as *generalisations* where entities are replaced by variables [29]. The above *pattern* conteins two variables ?Peptide and ?Protein. When the *pattern* is instanciated, the variables will be replaced with entities. For example, for peptide with ID 1023927 the variable ?Protein will be replaced with the parent protein from which the peptide is derived, which is PRO’s Collagen alpha-1(I) chain (PR:P02452). It should be noted that a *pattern* (a.k.a. *axiom pattern*) does not necessarily coincide with the notion of ontology design pattern (see [29]). A *pattern* can also represent a set of OWL axioms. And thus an ontology’s class expressions or definitions can be easily obtained instanciating patterns.

The description of proteins in the PxO follows guidelines for the PRO [18] and it uses the RO object properties ‘has function’ to relate a protein to its GO molecular_function, ‘has location’ to relate a protein to its location in a GO ‘cellular component’ and ‘participates in’ to relate a protein to the GO ‘biological process’ in which it may participate. Proteins are thus described in the PxO with the following axioms:



The aim in PxO is to describe protein types taken from Uniprot described by terms from the Gene Ontology according to PRO guidelines. Given the PRO protein Collagen alpha-1(I) chain (PR:P02452), the following two axioms are made by instanciating the above *pattern*



UniProtKB/Swiss-Prot [30] contain more than one hundred thousand protein records for metazoa (i.e. multicellular animals) that were reviewed, and manually annotated. Some of these proteins have isoforms, i.e. alternatives to the canonical sequence. The PxO is released currently with the PxO metazoa that contains 139 720 OWL protein classes (UniProtKB SwissProt and Isoform sequences), 4 591 OWL organism taxons, and with 89 846 OWL gene classes. Each type of OWL Class (protein, gene, and organism taxon) is generated using its own axiom pattern.

Both proteins and peptides have N-terminus- and C- terminus regions. Thus, axioms were introduced to refine PRO’s amino acid chain:



***Cleavage sites***: On the one hand, a cleavage site (CS) is part of a protein. On the other hand, a protein may have one or more CS. Therefore, two patterns were created. PxO is released currently with 16 273 OWL CS classes for known cleavage sites, which are associated with 5 084 distinct protein classes.

***Protease***: An equivalence axiom allows any protein with a GO annotation for peptidase activity(GO:0008233) or one of it’s children to be recognised as a protease by Proteasix. The axioms for representing proteases are as follows:



In the same vein, it is straight-forward to define a class such as exopeptidaseby using the GO annotation for exopeptidase activity (GO:0008238). Definitions for endopeptidase, aminopeptidase and carboxypeptidase are easily made by exploiting the GO’s catalytic activity hierarchy.

***Proteolysis***: Taking the GO’s proteolysis, it is feasible to create additional axioms to describe the biological process that has input participants of a substrate protein, a protease and has output participants proteolytic cleavage fragment



In the PxO there is a clear distinction between: a) observations of protease/CS combinations extracted from the literature (e.g. CutDB [8]); and b) prediction of cleavage based on protease’s cleavage site specificity from MEROPS [9] or exopeptidase’s cleavage site annotation assertions with the catalytic activity from BRENDA [10], which captures how likely it is for an amino acid to be present or absent in a certain position close to the CS. To represent this dichotomy, two classes were introduced: observed proteolysis and predicted proteolysis. To create their class definitions, the status of the proteolysis is indicated. Observed proteolysis is defined as:



In the PxO there are 20 229 observed proteolysis and 329 predicted proteolysis created from two patterns.

Annotation assertionsprovide the means to associate aditional information with an entity, like an exact synonym (oboInOwl:hasExactSynonym), a database cross reference (oboInOwl:hasDbXref), or a definition (IAO:0000115). When a protein taken from the UniProtKB has a MEROPS specificity matrix, annotation assertion axioms are used in PxO to represent this data. However, the probability for a protease to cleave a protein substrate is calculated outside of the PxO, although using the annotation properties and values in the PxO.

## PxO in use

There are two methods to find the protease classes in the PxO: 1) use an automated reasoner like ELK [31] to infer which proteins are proteases (see the Protease defined class in the previous section); or 2) use the SPARQL 1.1 query language [32] to create a SELECT query (Q0) that retrieves the OWL protein classes with a GO assertion for peptidase activity (GO:0008233) or any of it’s children. Likewise, using DL queries with ELK or SPARQL SELECT queries, the proteins that are endopeptidase, aminopeptidase or carboxypeptidase can be obtained.

The essence of the Proteasix algorithmis given below, which exploits the PxO ontology, and uses the competency questions CQ. The algorithm assumes that protease cleavage of substrate proteins is directed by short amino acid motifs, from two to eight amino acids of the type (Pn …)P1 - P1’(…Pn’), with the scissile bond between P1 and P1’ residues [5]. Residues towards the N-terminus of the substrate are on the non-prime side and numbered as P1 P2 P3 P4 and so on; while residues towards the C-terminus are on the prime side and numbered as P1’ P2’ P3’ P4’ and so on [33].

***STEP 1: User input*** — For each peptide, the end-user provides: a) the peptide identifier; b) the UniProt Accession Number (AC) or identifier (ID) of the parent protein from which the peptide is derived; c) the start amino acid position with respect to the parent protein’s sequence, i.e. P1’ of the N-terminus CS; and d) the end amino acid position with respect to the parent protein’s sequence, i.e. P1 of the C-terminus CS.

***STEP 2: Reconstruct N- and C-terminus CS*** — Each OWL protein class created from the UniProtKB (Swiss-Prot/TrEMBL) has among others the following annotation properties: oboInOwl:id for AC; oboInOwl: hasAlternativeId for ID; and PxO:hasSequence where the amino acid sequence (sequence for short) of the protein is stored. SPARQL query CQ2-2 in the Additional file 1 exemplifies how to obtain the amino acid sequence for protein P02768 (PR:P02768). Outside of PxO, and using the protein sequence, the peptide sequence for an input peptide is extracted, and the N- and C-terminus are reconstructed, i.e. eight amino acids or fewer if close to the beginning or end of the protein’s sequence. The output of this step is the creation for each input peptide of an OWL peptide class along with an OWL N-terminus class and an OWL C-terminus class.

***STEP 3: Observed cleavage*** — Using the class expression polypeptide region SubClassOf ’part of’ some ’amino acid chain’OWL CS classes and OWL protein classes are linked, and therefore, this class expression represents that *a CS is part of a protein*. An OWL CS class also has annotation properties for storing the CS sequence and the P1 and P1’ values. SPARQL query CQ2-1 in the Additional file 1 illustrates how to retrieve for protein P02768 (PR:P02768) the observed CS regions where the P1’ value is 25. To make the retrieval process more efficient, firstly, for each peptide, the sequence of its corresponding OWL N-terminus class and OWL C-terminus class are matched against the OWL CS class sequence. If successful, a more detailed match is triggered. A successful outcome of this step is an instantiation of the *axiom pattern*?peptide SubClassOf ’output of’ some (?proteolysis and (hasKnowledgeStatus some ’Observed status’)).

***STEP 4: Predicted cleavage*** — For the OWL N-terminus and C-terminus classes with sequences that remain unmatched after the previous step, a protein cleavage prediction is attempted. SPARQL query CQ4 in the additional file can be generalised by replacing the PRO’s protease P08253 (PR:P08253) for a parameter ?C, and thus, the results of the query will be the set of proteases for which prediction can be undertaken by exploiting the MEROPS cleavage site specificity matrix. The probability calculations are outside of PxO. Firstly, the probability of cleavage is estimated from the protease’s MEROPS specificity matrix [9] using a log-likelihood. If the probability is above the 99th percentile of the population distribution of all possible sequences, then the sequence is taken as statistically matched. A confidence level is then assigned to the matching, using levels from a simulation distribution of the matching step. Secondly, for the sequence’s that obtained a low/medium confidence level prediction or still have no prediction, BRENDA [10] exopeptidase’s catalytic information is used and a second prediction is attempted, assuming that after endopeptidase cleavage, and exopeptidase cuts the free extremity (i.e. C- or N- terminus CS). A successful outcome of this step is an instantiation of the axiom pattern*?peptide SubClassOf ‘output of’ some (?proteolysis and (hasKnowledgeStatus some ‘Predicted status’))*.

Further validation of the observed and predicted proteolysis (step 3 and 4) is accomplished by checking in PxO that: a) If the source organism for the protease and the substrate are the same; and b) if both the protease and the substrate are co-located.

***Positive example***: In the additional file, there is an SPARQL SELECT query (CQ3-2) that investigates whether the two above-mentioned conditions are met for PRO’s substrate Serum albumin (PR:P02768) and PRO’s protease 72 kDa type IV collagenase (PR:P08253). The query indicates that both protease and substrate protein may come from the same taxon Homo sapiens (NCBI:9606) and may have the following common co-locations: nucleus(GO:0005634); extracellular region(GO:0005576); extracellular space(GO:0005615). This is a positive corroboration of co-location. Indeed, there is evidence that the cleavage is observed.

***Negative example***: Reusing the SPARQL SELECT query (CQ3-2) with different protease Neutrophil elastase(PR:P08246) and substrate protein ATP-binding cassette sub-family A member 6(PR:Q8N139). The query indicates that both protease and substrate protein may come from the same taxon Homo sapiens (NCBI:9606), however no common co-locations are found. This reinforces the low confidence level status assigned to the prediction obtained.

## Discussion

The first version of Proteasix was agnostic as to species, location and function of the proteases and their substrate proteins. The PxO allows knowledge of the domain to be added to the Proteasix algorithm. The PxO allows Proteasix to add semantics to its data such that the algorithm can check proteases for their function and both protease and substrate for species and location, as well as making the data reliably queriable. The aim here is two-fold: first, separating operational knowledge from domain knowledge; this will enable update and expansion of the knowledge component with relative ease. Second, the aim is to allow Proteasix and human users to check the validity of Proteasix results. These results may not be improved due to the use of PxO, but may be interpreted with more confidence.

The bulk of the PxO has been developed through re-use of other ontologies and these were ontologies mainly from the OBO Consortium. In doing so we have committed to the ontological viewpoint of those ontologies. We have not substantially changed those ontologies, except to extend, as it makes interoperability with other OBO based applications harder, as well as update and maintenance more difficult. This commitment does, however, come at a potential cost. Like any commitment, making one commitment excludes others. The OBO, like most ontologies, are not without their controversies. The GO, in particular, has long had its critics both on ontological and logical grounds [34, 35] There is debate about whether the ‘molecular function’ in the GO is ontologically a function or a finer grained process than the GO’s ‘biological process [34]. The GO has also been criticised for inconsistency in its modelling and lack of constraints that allow automated reasoning to be applied more effectively [35, 36]. In the PxO we have taken these aspects into account with Proteasix’s application needs and resources, and have decided that commiting to the OBO is an appropriate choice; this decision will be kept under review.

There is much further work to be done in the PxO. Currently, we only incorporate cannonical and isoform information from the UniProtKB, which also contains much information about sequence variants. Inclusion of this may improve the analysis done by Proteasix. The Gene Ontology annotations used in describing proteins can be accompanied with evidence codes [13], and therefore, taking into account with what confidence annotations are made may also improve the utility of the PxO in Proteasix. Another line of work is to map the cleavage to the peptidase family instead of mapping the cleavage to an individual enzyme. A protein is typically represented as having many functions, in many locations and being involved in many biological processes. At present which functions, in which process and in which location is not represented. As this kind of representation emerges it will be adopted in PxO and may contribute to the accuracy of predictions made in Proteasix.

The PxO has its limitations in addition to those indicated by the future work. The PxO, like most ontologies, is limited by the state of knowledge in its domain, which for PxO is large. That confirmatory information on a protease protein interaction is not found does not mean it cannot occur. The knowledge in the literature is much greater than that in ontological form. Nevertheless, if PxO can help give confidence to Proteasix’s predictions then it is a help.

A further limitation comes in the ability to predict cleaveage sites in Proteasix. Proteases exhibit varying binding affinities for amino-acid sequences, ranging from strict restriction to one or few critical amino-acids in given positions, to generic binding with little discrimination between different amino acids. The MEROPS database [9] lists such information. When available, MEROPS specificity weight matrices were added to PxO. The MEROPS specificity matrix shows how frequently each amino acid occurred at each position in the cleavage site. Matrices were further transformed into Probability Matrices, by dividing the number of occurrences for each amino acid in each position with the total number of observations. It has been acknowledged that to be able to study peptidase specificity and make predictions about where in a protein cleavage might occur, at least 40 cleavages in substrates are required [33] and/or a minimal 10 times enrichment for one amino acid should be observed in at least one position of the CS. Hence, the availability of MEROPS peptidase specificity data is a hard limitation of the current approach, which is a limitation inherent to experimental science. However, the predictions of Proteasix will improve as the body of evidence increases.

This research work was done for the *sysVASC* project, and so the emphasis is on Metazoa, where the organisms human, mouse, and rat are the main focus. Despite the large amount of work still to do in the PxO, the PxO nevertheless is a rich ontology supporting peptide analysis that has been enabled by re-using the ontologies produced by the OBO Consortium; the PxO has partitioned these ontologies based on the task they need to support and enriched them axiomatically on the same basis. As a result Proteasix can better support the prediction of proteases implicated in the production of peptides and the consequent elucidation of biological mechanisms.

## Additional file

Additional file 1 PxO Metazoa ontology: Ontology metrics; ELK reasoner times; and SPARQL queries execution times. (PDF 141 kb)
