# Supplementary material for: The Proteasix Ontology
Source: J Biomed Semantics. 2016 Jun 4;7:33. doi: 10.1186/s13326-016-0078-9 (PMC4893253; doi:10.1186/s13326-016-0078-9)
Supplement: Additional file 1 — PxO Metazoa ontology: Ontology metrics; ELK reasoner times; and SPARQL queries execution times. (PDF 141 kb) [file 13326_2016_78_MOESM1_ESM.pdf]

The Proteasix Ontology – Additional file 1

Ontology metrics

Table 1 – Ontology metrics for PxO Metazoa

**Axioms**	3 879 562
**Logical Axiom count**	1 700 233
**Class count**	313 174
**Object property count**	25
**SubClassOf axiom count**	1 699 936
**EquivalentClass axiom count**	285
**DL expressivity**	ALEH+

ELK reasoner times

Using a MacBook Pro Retina with 2.8 GHz Intel Core i7 and 16GB of RAM memory, the mean time for executing the classification three times was 51 seconds.

SPARQL 1.1. SELECT queries using JENA ARQ[1]

Q0 – A SPARQL 1.1 SELECT query that retrieves the OWL protein classes with a GO assertion for peptidase activity (GO:0008233) or any of it's children.

```
SELECT * FROM <file:./InOWL/PxOmetazoa.owl> WHERE {
      ?C rdfs:subClassOf+ obo:GO_0008233 .

      ?x rdf:type owl:Class ;
         rdfs:subClassOf [ a owl:Restriction ;
         owl:onProperty obo:RO_0000085 ;
         owl:someValuesFrom ?C ] .
}
```

Competency questions for PxO

CQ 1 – What are the known protease and their target cleavage sites (observed and/or predicted)?

One of the results for query Q0 is P08253 (obo:PR_P08253).

CQ1-1 The following SPARQL 1.1 SELECT query retrieves the cleavage site regions for which there are an observed proteolysis that has as input P08253 (obo:PR_P08253).

```
SELECT ?C FROM <file:./InOWL/PxOmetazoa.owl> WHERE {

      ?x rdfs:subClassOf obo:GO_0006508 .

        ?x rdf:type owl:Class ;
           rdfs:subClassOf  [ a owl:Restriction ;
           owl:onProperty galen:hasKnowledgeStatus ;
           owl:someValuesFrom PxO:ObservedStatus  ] .

        ?x rdf:type owl:Class;
            rdfs:subClassOf  ?y1 .

        ?y1 rdf:type owl:Restriction ;
        owl:onProperty  obo:RO_0002233 ;
        owl:someValuesFrom  obo:PR_P08253  .
```

[1] The namespace prefix bindings are omitted in the SPARQL queries

```
        ?x rdf:type owl:Class;
            rdfs:subClassOf  ?y2 .

        ?y2 rdf:type owl:Restriction ;
        owl:onProperty  obo:RO_0002233 ;
        owl:someValuesFrom  ?z  .

        ?z rdf:type owl:Class;
        owl:intersectionOf [ list:member ?el ] .

        ?el owl:onProperty obo:BFO_0000051 ;
            owl:someValuesFrom ?C .

      ?C rdfs:subClassOf PxO:CleavageSiteRegion .
 }
```

CQ1-2 The following SPARQL 1.1 SELECT query investigates if there is a predicted proteolysis that has as input P08253 (obo:PR_P08253).

```
SELECT * FROM <file:./InOWL/PxOmetazoa.owl> WHERE {

      ?x rdfs:subClassOf obo:GO_0006508 .

        ?x rdf:type owl:Class ;
           rdfs:subClassOf  [ a owl:Restriction ;
           owl:onProperty galen:hasKnowledgeStatus ;
           owl:someValuesFrom PxO:PredictedStatus  ] .

        ?x rdf:type owl:Class;
            rdfs:subClassOf  ?y .

        ?y rdf:type owl:Restriction ;
        owl:onProperty  obo:RO_0002233 ;
        owl:someValuesFrom  obo:PR_P08253  .
 }
```

If there are results for the above-mentioned query, the amino acid sequence of the cleavage site region for a protein substrate must be provided to calculate the probability of the cleavage to happen. Although the probability calculations are outside of PxO, it is necessary to execute CQ2-2 as well as CQ4 (see both queries below).

CQ 2 – For a given peptide and protein from which it was derived, what are the cleavage sites that led to its production and is it the product of observed or predicted proteolysis?

Let's assume that we have the following input peptide 89325 (Peptide-ID) that derives from P02768 (obo:PR_P02768), where the start amino acid position is 25 and the end amino acid position is 44. Both numbers with respect to the sequence of P02768.

CQ2-1 The following SPARQL 1.1 SELECT query retrieves for protein P02768 (obo:PR_P02768) the observed CS regions

```
SELECT * FROM <file:./InOWL/PxOmetazoa.owl> WHERE {

      ?C rdfs:subClassOf+ PxO:CleavageSiteRegion;
         PxO:hasP1primeCleavageSitePosition "25"^^xsd:positiveInteger  .

        obo:PR_P02768 rdf:type owl:Class;
           rdfs:subClassOf  [ a owl:Restriction ;
           owl:onProperty obo:BFO_0000051 ;
           owl:someValuesFrom ?C  ] .
 }
```

For the prediction, this goes beyond the PxO ontology on its own. We need a) to get the protein substrate sequence out of a query to the ontology; and b) extract part of the sequence and do the probability calculations. Both operations are being done outside the PxO ontology.

CQ2-2 The following SPARQL 1.1 SELECT query obtains the amino acid sequence for P02768 (obo:PR_P02768)

```
SELECT * FROM <file:./InOWL/PxOmetazoa.owl> WHERE {
        obo:PR_P02768 rdf:type owl:Class;
        PxO:hasSequence  ?y .                         }
```

CQ 3 – What are the function, species and cellular location for both proteases and their substrate proteins?

CQ3-1 The following SPARQL 1.1 SELECT query obtains the GO annotations from GO sub-ontology *molecular function* for P08253 (obo:PR_P08253).

```
SELECT * FROM <file:./InOWL/PxOmetazoa.owl> WHERE {
        obo:PR_P08253 rdf:type owl:Class;
           rdfs:subClassOf  [ a  owl:Restriction ;
           owl:onProperty      obo:RO_0000085 ;
           owl:someValuesFrom  ?y ] .

      ?y rdfs:subClassOf+ obo:GO_0003674;
           rdfs:label  ?lby .
 }
```

Note: The above query can obtain the GO annotations from GO sub-ontology *molecular function* for P02768 (obo:PR_P02768) by replacing in the query obo:PR_P08253 for obo:PR_P02768.

CQ3-2 A SPARQL 1.1 SELECT query that for substrate P02768 (obo:PR_P02768) and protease P08253 (obo:PR_P08253) looks a) if the source organism for the protease and the substrate are the same; and b) if both the protease and the substrate are co-located.

```
SELECT * FROM <file:./InOWL/PxOmetazoa.owl> WHERE {

        obo:PR_P08253 rdf:type owl:Class;
           rdfs:subClassOf  [ a  owl:Restriction ;
           owl:onProperty      ?p ;
           owl:someValuesFrom  ?y ] .

       obo:PR_P02768 rdf:type owl:Class;
           rdfs:subClassOf  [ a  owl:Restriction ;
           owl:onProperty    ?p    ;
           owl:someValuesFrom  ?y ] .

      ?y rdfs:label  ?lby .

      FILTER (?p =  obo:RO_0001025 || ?p = pr:only_in_taxon)
 }
```

CQ 4 – For a given protease, what are its cleavage site specificity?

One of the results for query Q0 is P08253 (obo:PR_P08253).

Although the probability calculations are outside of PxO, some proteases have MEROPS cleavage site specificity matrix. The specificity matrix shows how frequently each amino acid has been found to occur in each position around the scissile bond (for more details, see *Specificity logos and matrices* at https://merops.sanger.ac.uk/about/special_features.shtml)

CQ4 A SPARQL 1.1 SELECT query that for protease P08253 (obo:PR_P08253) retrieves the probabilities calculated from MEROPS cleavage site specificity matrix

```
SELECT * FROM <file:./InOWL/ PxOmetazoa.owl> WHERE {

 obo:PR_P08253 rdf:type owl:Class;
                 ?p  ?y .

 ?p rdfs:subPropertyOf PxO:hasProbabilityForSpecificityMatrixForCleavageSitePosition.
}
```

CQ 5 – Given an amino acid, what are its biochemical properties?

The biochemical properties assigned to amino acids were modelled by placing restrictions on object property expressions.

CQ5 A SPARQL 1.1 SELECT query that investigates the biochemical properties of amino acid Glycine (obo: CHEBI_15428).

```
SELECT * FROM <file:./InOWL/PxOmetazoa.owl> WHERE {

        obo:CHEBI_15428 rdf:type owl:Class;
           rdfs:subClassOf  [ a  owl:Restriction ;
           owl:onProperty      ?p ;
           owl:someValuesFrom  ?y ] .
 }
```

CQ 6 – For a protease predicted to have generated a peptide, what are its function and the processes in which it is known to participate?

One of the results for query Q0 is P08253 (obo:PR_P08253).

CQ6 A SPARQL 1.1 SELECT query that retrieves the GO annotations for protease P08253 (obo:PR_P08253) from two GO namespaces `molecular function`; and `biological process`.

```
SELECT * FROM <file:./InOWL/PxOmetazoa.owl> WHERE {
    {
      obo:PR_P08253 rdf:type owl:Class;
           rdfs:subClassOf  [ a  owl:Restriction ;
           owl:onProperty      ?p ;
           owl:someValuesFrom  ?y ] .

      ?y rdfs:subClassOf+ obo:GO_0003674;
           rdfs:label  ?lby .

      FILTER (?p =  obo:RO_0000085)
   } UNION {
        obo:PR_P08253 rdf:type owl:Class;
           rdfs:subClassOf  [ a  owl:Restriction ;
           owl:onProperty      ?p ;
           owl:someValuesFrom  ?y ] .

      ?y rdfs:subClassOf+ obo:GO_0008150;
           rdfs:label  ?lby .
      FILTER (?p =  obo:RO_0000056)
  }
}
```

Execution times of SPARQL queries for PxO Metazoa using JENA ARQ

Table 2 shows the mean time for executing the above-mentioned SPARQL three times using a MacBook Pro Retina with 2.8 GHz Intel Core i7 and 16GB of RAM memory.

Table 2 – Mean time for executing the same SPARQL query three times

**Query**	**Time**
**Q0**	248 seconds (4.1 minutes)
**CQ1-1**	203 seconds (3.4 minutes)
**CQ1-2**	202 seconds (3.4 minutes)
**CQ2-1**	217 seconds (3.6 minutes)
**CQ2-2**	203 seconds (3.4 minutes)
**CQ3-1**	207 seconds (3.4 minutes)
**CQ3-2**	213 seconds (3.5 minutes)
**CQ4**	212 seconds (3.5 minutes)
**CQ5**	199 seconds (3.3 minutes)
**CQ6**	207 seconds (3.4 minutes)
